# Impact of stopping burosumab treatment at the end of skeletal growth in adolescents with X-linked hypophosphatemia (XLH)

**DOI:** 10.1016/j.bonr.2024.101819

**Published:** 2024-11-24

**Authors:** Charlotte Jarvis, Renuka Ramakrishnan, Poonam Dharmaraj, Talat Mushtaq, Sanjay Gupta, Angela Williams, Angela J. Rylands, Helen Barham, Annabel Nixon, Suma Uday

**Affiliations:** a Alder Hey Children's NHS Foundation Trust, Liverpool L12 2AP, UK; b Leeds Teaching Hospitals NHS Trust, Leeds LS9 7TP, UK; c Hull University Teaching Hospitals NHS Trust, HU3 2JZ, UK; d Kyowa Kirin International PLC, Marlow SL7 1HZ, UK; e Chilli Consultancy, Salisbury SP1 1JS, UK; f Birmingham Women's and Children's NHS Foundation Trust, Birmingham B15 2TG, UK

**Keywords:** X-linked hypophosphatemia, Burosumab, Case series

## Abstract

Many adolescents with X-linked hypophosphatemia (XLH) currently have to stop treatment with burosumab at the end of skeletal growth. We describe the experience of a cohort of adolescents with XLH before, during, and after stopping burosumab (median treatment duration 37.5 months). Improvements in serum phosphate, pain, mobility, function, and quality of life noted during burosumab treatment were reversed after treatment cessation. Further real-world data are needed to explore the value of uninterrupted burosumab treatment in adolescents.

## 1 Introduction

X-linked hypophosphatemia (XLH) is a rare, genetic, progressive, lifelong phosphate-wasting disorder caused by loss-of-function mutations in the *PHEX* (phosphate-regulating endopeptidase homologue, X-linked) gene, leading to excess circulating levels of fibroblast growth factor 23 (FGF23) (Beck-Nielsen et al., 2019). This leads to renal phosphate wasting and impaired production of 1,25-dihydroxy vitamin D, with compromised intestinal absorption of phosphate. Chronic hypophosphatemia and dysregulation of bone metabolism during childhood can compromise skeletal development, leading to delayed and disproportionate growth and short stature (Beck-Nielsen et al., 2019; Chen et al., 2018; Cremonesi et al., 2014; Orlando et al., 2022). Children with XLH often experience skeletal pain (Beck-Nielsen et al., 2019; Skrinar et al., 2019) and deformity that results in gait abnormality and may require orthopedic surgery. Children also experience muscle weakness and pain (Romagnoli et al., 2022), and dental mineralization is often impaired (Skrinar et al., 2019). Children with XLH have been reported to have difficulty attending school and participating in usual childhood activities (Ferizovic et al., 2020). Children, adolescents, and adults with XLH experience a substantial burden in terms of health-related quality of life (HRQL) and psychological and emotional wellbeing, including negative social experiences, low self-esteem, frustration, depression, and anxiety about the future (Cheung et al., 2021).

Conventional therapy for XLH for many years has been supplementation with oral phosphate and active vitamin D analogues; however, the therapeutic benefit is variable, the treatment regimen is burdensome, and patients often experience gastrointestinal side-effects. Adherence to conventional therapy is often poor (Linglart et al., 2014). Patients are also at risk of long-term adverse metabolic effects such as hypercalciuria, nephrocalcinosis, and hyperparathyroidism (Linglart et al., 2014; Skrinar et al., 2019).

Burosumab is a fully human monoclonal antibody that binds to and inhibits the activity of excess circulating FGF23 (Insogna et al., 2018). In a phase 3 trial in children aged 1–12 years, 64 weeks' treatment with burosumab improved growth, markers of bone biochemistry, and radiological rickets severity compared with conventional therapy (Imel et al., 2019). Burosumab treatment has been shown to improve patient-reported pain interference, fatigue, and HRQL in children (Padidela et al., 2021), and significant improvements in physical function, stiffness, pain, and fatigue have been reported in adults with XLH treated with burosumab (Insogna et al., 2018; Portale et al., 2019).

The UK National Institute for Health and Care Excellence (NICE) recommended burosumab in 2018 for the treatment of XLH in children aged ≥1 year with radiographic evidence of bone disease and in young people with growing bones. However, evidence to support use of burosumab in adolescents beyond the end of skeletal growth (EoSG) was considered lacking. Thus, adolescents in England and Wales had to stop treatment at the end of longitudinal growth. NICE published its final guidance recommending the use of burosumab in adults in July 2024 and also updated its guidance in September 2024 recommending burosumab for the treatment of children aged 1–17 years. However, adolescents in other European countries may have difficulty accessing burosumab treatment and face interruption to treatment because the reimbursed/funded indication (depending on jurisdiction) is narrower than the licensed indication (Mughal et al., 2023).

This retrospective multicenter case series explores the impact of stopping burosumab treatment at EoSG in adolescents with XLH. It was conducted before NICE updated its recommendations for use of burosumab in older children and adults.

## 2 Methods

### 2.1 Study design

This is a multicenter retrospective descriptive case series.

### 2.2 Case selection

Patients attending four of the 15 hospitals in England that are currently licensed to prescribe burosumab for pediatric use over the previous 12–24 months were screened by the treating clinicians. To be eligible, patients had to have received at least 6 months' burosumab treatment for XLH and to have stopped treatment at EoSG, as determined by the treating physician based on slowing of growth velocity or radiographic evidence of growth plate closure. Case selection was not related to age at start of burosumab treatment or to treatment received before or after burosumab.

### 2.3 Data collection

To standardize the information captured, the study sponsor designed case study report forms covering medical and treatment history, biochemistry, and radiology results, and the clinicians' observations of the patients' experience of XLH before, during, and after burosumab treatment. The treating physicians completed the case study report form with information from the patients' health records and their recollection of patient and family reports during clinical visits.

### 2.4 Reporting

The case studies described here were developed from the information in the case study report forms and reviewed by the treating physicians for accuracy and anonymity. Note that phrases in quotes are from the case report forms and may not be verbatim from the patients or families.

Medians and ranges for phosphate values were calculated from single values for each patient at each stage of treatment.

### 2.5 Consent

Written informed consent was obtained from patients' guardian/carers by the treating physicians.

## 3 Results

Eight adolescents with XLH (3 male, 5 female) were eligible for inclusion. The patients started burosumab treatment at 9–16 years of age and the median duration of treatment was 37.5 months (range 22–53 months). Details of diagnosis and treatment history are presented in Table 1.

**Table 1:** Diagnosis and treatment history.

Case	Sex	Age at XLH diagnosis	Basis of XLH diagnosis		Age at start of conventional therapy	Burosumab treatment				Subsequent treatment^a^
			Family history	*PHEX* test confirmation		Age at start	Duration	Age at end	Reason for stopping	
1	F	1 y 9 m	Yes	No	1 y 6 m	14 y 1 m	2 y 5 m	16 y 6 m	RGV	None (tolerability)
2	F	2 y 1 m	Yes	No	2 y 1 m	10 y 10 m	3 y 8 m	14 y 6 m	GPC	OP + AC after 3 y because of pain
3	M	5 y 6 m	No	Yes	NR	13 y	4 y 4 m	17 y 4 m	RGV	OP + AC (timing not reported)
4	F	3 y 1 m^b^	No	Yes	3 y	12 y 5 m	2 y 4 m	14 y 9 m	GPC, RGV	None (inconvenience, stigma)
5	F	5 m	Yes	Yes	10 m	9 y 3 m	3 y 11 m	13 y 2 m	GPC	None (reason not reported)
6	M	1 y 6 m	Yes	Yes	3 y	~16 y	2 y	17 y 10 m	GPC	OP + AC after 8 m because of pain
7	M	2 y	No	Yes	~2 y	14 y 9 m	2 y 7 m	17 y 4 m	RGV	OP + AC (timing not reported)
8	F	Soon after birth	Yes	Yes	Infancy	14 y 1 m	4 y 5 m	18 y 6 m	RGV, age 18 y	OP + AC after 4 m

AC, alfacalcidol; F, female; GPC, growth plate closure; M, male; m, month; NR, not reported; OP, oral phosphate; *PHEX*, phosphate-regulating endopeptidase homologue, X-linked, gene; RGV, reduced growth velocity; XLH, X-linked hypophosphatemia; y, year.

a Time of restarting CT is after stopping burosumab.

b Presented with varus deformity at age 2 y 6 m, which was treated as nutritional rickets from age 3 y 1 m on the basis of radiography and clinical chemistry.

### 3.1 Clinical chemistry

Median phosphate concentrations are presented in Table 2; individual clinical chemistry results are presented in Supplementary Table S1. Plasma phosphate levels were mostly below the local reference range when conventional therapy was stopped prior to starting burosumab treatment, increased to within or close to normal range at the end of burosumab treatment but decreased to below reference range after stopping burosumab treatment at EoSG.

### 3.2 Patients' experience before burosumab treatment

All patients had been receiving conventional therapy before switching to burosumab. The age at starting treatment varied from infancy (patient #8) up to 3 years of age (#4, 6). Gastrointestinal side-effects, particularly diarrhea, were specifically reported for two patients (#1, 8). One physician reported that their patient did not like taking conventional therapy (#8).

**Table 2:** Median serum phosphate levels before, during, and after burosumab treatment.

	n	Median	Range
Before	7	0.59	0.54–0.99
During	8	0.87	0.76–1.12
After	6	0.60	0.48–0.66

Medians are calculated from single values for each patient at each stage of treatment; where several values were available, “before” values are those closest to starting burosumab, “During” are values closest to the end of burosumab treatment, and “after” values are the furthest after stopping treatment.

Six of the patients (#1, 2, 4, 6, 7, 8) were reported to have developed skeletal deformities during childhood, five of whom (#1, 4, 6, 7, 8) had undergone one or more orthopedic surgical procedures to correct deformities (Table 3).

**Table 3:** Orthopedic surgical history before, during, and after burosumab treatment.

Case study	Before burosumab	During burosumab treatment	After stopping burosumab
1	Surgery to correct genu varum (both legs) at age 7 y Plates removed after 2 y Further surgical alignment at age 11 y	Osteotomy, right leg, at age 14 y (B treatment started at age 14 y 1 m) Patellar instability plus removal of plate Right distal femoral medial closing weight osteotomy Quicker recovery reported	None
2	None	None	None
3	None	Surgery to correct genu varum planned at end of skeletal growth but delayed by Covid pandemic	
4	Plate insertion at age 5 y Frames to correct tibial deformity for 6 m at age 9 y Plate insertion at age 12 y	Not reported	Not reported
5	None	None	None
6	Five procedures; details not reported	Plate removal	Not reported
7	Lateral plates, distal femur and proximal tibia, both lower limbs, to correct shape at age 5 y Plates and screws removed after 18 m	Not reported	Not reported
8	Plates bilateral medial aspect of distal femur and bilateral proximal aspect of medial tibia at age 10 y Metalwork removed after 12 m Plates inserted to control progression of deformity at age 13 y	Right distal femur, proximal and distal tibia external fixator-assisted deformity correction and internal fixation at age 14 y (just after starting B) Metalwork removed after 11 m and 3 y 4 m	Not reported

B, burosumab; m, month; y, year.

Five patients were reported to experience pain in the legs, which impaired mobility (#6) or required pain relief to take part in sporting activities (#7). Patients were reported to have gait disturbance (#1, 4, 8) and impaired or restricted mobility (#1, 6, 8), and were unable to participate in physical education (PE) in school (#2, 4, 8). One patient was reported to have had multiple falls and had difficulty keeping up with peers (#8). Four patients were reported to experience fatigue and tiredness (#1, 4, 6, 8). One was reported to experience pain and stiffness in the morning, making it difficult to start the day (#8).

Whilst there was no formal assessment of quality of life (QoL) or mental wellbeing, three patients were considered by their physicians to have impaired QoL because of poor mobility and pain or the gastrointestinal side-effects of conventional therapy (#1, 6, 8). Physicians also described patients as feeling left out of activities because of pain/mobility difficulties and not engaging with consultation.

### 3.3 Patients' experience during burosumab treatment

Six patients had decreased pain while on burosumab (#1, 3, 4, 6, 7, 8). Patients 1, 7, and 8 had painful legs and joints before taking burosumab but had less frequent, or no, pain while taking burosumab and required less pain relief. Improvements in skeletal deformities were reported for three patients (#2, 5, 8). Two underwent orthopedic surgery (other than plate removal) soon after starting burosumab; one reported quicker recovery compared with before burosumab treatment (#1).

Most patients were reported to have improved joint flexibility (#1), mobility (#1, 7), and function (#1, 6, 7, 8) while taking burosumab, such as being able to walk further (#1, 4), being able to participate in school/college-based PE/sport (#1, 4, 7), being able to attend school regularly (#8) or being able to take on physical work (#7). Patients were also described as having more energy (#4, 6, 7) and to be less tired, better at getting up in the morning, and “brighter” (#8).

Marked improvements in mental wellbeing and QoL were noted. Patients were described as having much improved QoL (#2, 4, 6), “feeling good” on treatment (#1), and being “more enthusiastic” (#6). One (#6) reported a “transformation in QoL” and that they had “never felt so energetic and pain-free”.

### 3.4 Patients' experience after stopping burosumab treatment

The age at end of treatment ranged from 13 years 2 months to 18 years 6 months in the five girls, and from 17 years 4 months to 17 years 10 months in the three boys. Reasons for stopping burosumab were recorded as reduced growth velocity (#1, 3, 4, 7, 8), growth plate closure on radiograph (#2, 4, 5, 6), and age 18 (#8) (Table 1).

All but two patients (#1, 2, 4, 6, 7, 8) reported that pain returned within a few months after stopping burosumab treatment or was worse than during treatment and required increased use of analgesics. Most patients reported reduced mobility (#1) or physical function (#1, 2, 4, 8) after stopping burosumab treatment compared with during treatment, although some maintained at least some of the benefit seen during treatment (#1, 4, 6, 7). Four patients reported increased tiredness/fatigue (#1, 2, 6, 8). Increased pain and decreased mobility compromised patients' ability to participate in PE, physical activity (#4), work/college (#2, 6, 7, 8), and social activities (#7). One (#8) reported difficult walking, feeling weak and experiencing repeated falls.

For five patients (#1, 2, 3, 6, 7), stopping burosumab treatment was reported to have had a marked impact on their mental wellbeing, especially having experienced notable improvements in pain, function, and participation in daily activities whilst receiving burosumab. For one patient (#3), stopping treatment coincided with a critical stage of education and the change in treatment had a “marked impact” on the patient's morale and college attendance.

One patient (#8) was reported to be “always tired”, started getting migraine headaches, and struggled with energy and motivation. The patient described “coming down like a crash after 3 weeks of no treatment” and that on burosumab injections “she could live as an able person but this been taken away”.

Five patients returned to conventional therapy, but rarely immediately, and in some cases only when they started experiencing pain (#2, 6). One (#4) wanted to avoid the inconvenience and stigma and another had previously experienced gastrointestinal intolerance on conventional therapy (#1). The mother of one patient (#7) commented on the inconvenience of frequent phosphate dosing.

The physicians noted patients' families' concerns and frustrations about stopping burosumab treatment and were keen to explore ways to continue burosumab having observed the benefits of treatment (#1, 4, 7, 8).

## 4 Discussion

This case series describes the impact of stopping burosumab treatment in eight adolescents with XLH who were aged 9–16 years at the start of burosumab treatment and received treatment for a median of 37.5 months. Burosumab treatment improved the physical manifestations of XLH, such as pain, mobility, and fatigue, consistent with findings in clinical studies in children and adults (Briot et al., 2021; Padidela et al., 2021). During burosumab treatment the adolescents were better able to participate in school, PE/sport, and work than before treatment, thus achieving broader benefit than symptom relief, which is consistent with a 2022 consensus statement that highlights the importance of considering the social and psychological, as well as physical, impacts of XLH (Trombetti et al., 2022). However, stopping burosumab treatment heralded a return of pain and other symptoms for the patients in this case series, with a marked detrimental effect on daily activities, mental wellbeing, and QoL. Notably, patients and their families were reported to be frustrated and angry at having to stop a treatment that they considered to have improved their daily lives.

A European Expert Practice Exchange Group recommends that burosumab is started as early as possible (from 1 year of age), and is continued through adolescence, as maintaining adequate serum phosphate is likely to be beneficial for long-term bone health and may halt disease progression (Mughal et al., 2023). Importantly, the skeleton continues to mature and acquire bone mass beyond growth plate closure (Mughal et al., 2023). While the recent change in NICE guidance means that children in the UK can now continue burosumab treatment through adolescence and into adulthood, in some jurisdictions, adolescents currently have to stop treatment once skeletal growth is complete, which may be detrimental.

The need for continued treatment to maintain the benefits of burosumab has been demonstrated in a recent study in adults – an exploratory analysis based on seven patients found that some of the benefits of burosumab treatment were lost when burosumab treatment was interrupted for up to 15 months (although not to pretreatment levels); however, benefits returned slowly over 36 weeks or longer when treatment was resumed (Kamenicky et al., 2023). Furthermore, a case study of five adolescents (mean age 15.4 ± 1.5 years) who received burosumab treatment until 18 or 19 years of age (12–48 months' treatment) reported that discontinuation of burosumab treatment in four patients was associated with a decrease in serum phosphate, reversal of other improvements in clinical chemistry, and progressive worsening of patient-reported outcomes (pain, stiffness, and physical function), whereas these consequences were not seen in one patient who was still receiving treatment (Baroncelli et al., 2024). Thus, it seems likely that adolescents who stop burosumab treatment would experience benefits if they resume treatment as an adult, although this would need to be explored in clinical studies. The duration of treatment interruption will vary, however, and is likely to be longer in girls than boys – the mean age at end of treatment in the current case series was 15 years in the five girls (range 13 years 2 months to 18 years 6 months) and 17 years 1 month in the three boys (17 years 4 months to 17 years 10 months). Depending on the criterion used for EoSG, girls might be expected to stop burosumab treatment earlier than boys, potentially facing increased pain, worse mobility, and compromised mental health at a younger age and for a longer period.

This is the first published study that reports on the impact of stopping burosumab treatment specifically at the end of skeletal growth. The findings should be interpreted with caution because of the limitations inherent in this type of study. Data were obtained retrospectively from clinical records, and the detail of reporting varied. While standardized case study report forms were used, not all symptoms of interest were available from clinical records. Absence of reporting does not necessarily mean absence of a symptom if a physician did not record it at the time of the clinical visit. In addition, patient experiences are provided in the words of the treating physicians as retrospectively reported narratives rather than recorded directly from patients and families verbatim. Quantitative HRQL data are not routinely collected in clinical practice and therefore were not available for this case series; however, HRQL data are currently being collected in the International XLH Registry (described below). Blood chemistry data were limited and varied in terms of timing, number of measurements, and local laboratory reference ranges. However, improvements in bone biochemistry with burosumab treatment has been demonstrated in clinical trials.

Real-world data from adolescents who receive continuous treatment with burosumab may provide a useful comparison with those whose treatment is stopped or interrupted, and also contribute to the evidence base for burosumab treatment in adolescents with XLH. Such data are being collected via the International XLH registry (NCT03193476) established in 2017 to characterize the treatment, burden of disease, disease progression, and long-term outcomes of XLH in patients of all ages, and in the XLH Disease Monitoring Program (NCT03651505) – a global, prospective, multicenter, longitudinal, long-term outcomes program that is characterizing the presentation and progression of XLH (Ariceta et al., 2023; Padidela et al., 2020) – and in the SUNFLOWER longitudinal, observational cohort study (NCT03745521), which started in 2018 and plans to follow 160 patients with XLH in Japan and Korea for up to 5 years.

In conclusion, this case series of eight adolescents reports a consistent pattern of restored serum phosphate levels, reduced pain, and improved function and wellbeing during burosumab treatment. Serum phosphate levels decreased after stopping burosumab at EoSG, with a concomitant worsening of symptoms, which had a marked impact on patients' physical and mental wellbeing. Real-world data, focusing on patient-relevant outcomes, are needed to explore the value of uninterrupted burosumab treatment in adolescent patients, to support access to this treatment for these patients.

The following is the supplementary data related to this article.

Supplementary Table S1 Individual laboratory values reported before, during, and after burosumab treatment Supplementary Table S1

## CRediT authorship contribution statement

**Charlotte Jarvis:** Writing – review & editing, Methodology, Investigation, Data curation, Conceptualization. **Renuka Ramakrishnan:** Writing – review & editing, Methodology, Investigation, Data curation, Conceptualization. **Poonam Dharmaraj:** Writing – original draft, Methodology, Investigation, Data curation, Conceptualization. **Talat Mushtaq:** Writing – review & editing, Methodology, Investigation, Data curation, Conceptualization. **Sanjay Gupta:** Writing – review & editing, Methodology, Investigation, Data curation, Conceptualization. **Angela Williams:** Writing – review & editing, Supervision, Resources, Methodology, Funding acquisition. **Helen Barham:** Writing – original draft, Methodology. **Annabel Nixon:** Writing – review & editing, Writing – original draft, Methodology. **Suma Uday:** Writing – review & editing, Methodology, Investigation, Data curation, Conceptualization.

## Declaration of competing interest

CJ has received consulting fees from Kyowa Kirin, and support for attending meetings and/or travel from Kyowa Kirin and Inozyme. RR has received support for the present manuscript and payment or honoraria from Merck and Kyowa Kirin. PD has received payment for an educational event as an invited speaker, and support for attending meetings and/or travel from Kyowa Kirin. TM has received consulting fees and payment or honoraria from Novo Nordisk and Kyowa Kirin, support for attending meetings from Novo Nordisk and Pfizer, and is Secretary of the British pediatric and Adolescent Bone Group and Chair Elect of the British Society for pediatric Endocrinology and Diabetes. SG has received support for meeting attendance from Kyowa Kirin. AR and AW are employed by Kyowa Kirin. AN and HB undertake work for Chilli Consultancy; the company has received payment from Kyowa Kirin to support the development of this manuscript. SU has received a speaker fee and travel grant from Kyowa Kirin, is an advisory board member for Kyowa Kirin, and is an expert panel member for the International Hypophosphataemia Network.

## Data Availability

The authors do not have permission to share data.
