# Supplementary material for: Impact of stopping burosumab treatment at the end of skeletal growth in adolescents with X-linked hypophosphatemia (XLH)
Source: Bone Rep. 2024 Nov 24;24:101819. doi: 10.1016/j.bonr.2024.101819 (PMC11638637; doi:10.1016/j.bonr.2024.101819)
Supplement: Supplementary Table S1 — Individual laboratory values reported before, during, and after burosumab treatment [file mmc1.docx]

**Impact of stopping burosumab treatment at the end of skeletal growth in adolescents with X-linked hypophosphatemia (XLH)**

Jarvis et al

Supplementary Table S1 Individual laboratory values reported before, during, and after burosumab treatment

| # | **Phosphate**  **(mmol/L)** | | | **25-hydroxyvitamin D**  **(nmol/L)** | | | **Adjusted calcium**  **(mmol/L)** | | | **PTH**  **(pmol/L)** | | | **ALP**  **(IU/L)** | | |
| --- | --- | --- | --- | --- | --- | --- | --- | --- | --- | --- | --- | --- | --- | --- | --- |
|  | Before | During | After | Before | During | After | Before | During | After | Before | During | After | Before | During | After |
| 1 | *0.81–1.61* | *0.74–1.55* |  | *>50* | |  | *2.15–2.74* | |  | *1.1–6.9* | |  | *55–185* | *38–123* |  |
|  | **0.69** | **0.80** | NR | **38** | **15** |  | **2.37** | **2.19** | NR | **5.2** | **9.1** | NR | **409** | **109** | NR |
| 2 |  | *0.9–1.8* | *0.5–1.5* |  | *>50* |  |  |  | *2.20–2.60* |  | *13–29* | *12–65* |  | *35–50* | *25–90* |
|  | NR | **0.83** | **0.48** | NR | **19** | NR |  | **2.24** | **2.33** | NR | **85** | **70** | NR | **129** | **506** |
| 3 | *1.05–1.82* | | | *50–100* | | | *NR* | | | *1.5–7.6* | | | *115–471* | | |
|  | **0.99** | **1.13** | **0.6** | **63** | **45** | **59** | **2.2** | **2.33** | **2.3** | **6.2** | **6.4** | **7.4** | **314** | **163** | **115** |
| 4 | *0.9–1.8* | | *0.9–1.55* | *NR* | | | *NR* | *2.20–2.70* |  | *1.38–3.08* | | *1.6–6.9* | *65–240* | | *NR* |
|  | **0.46** | **0.82** | **0.66** | **57.9** | NR | NR | **2.26** | **2.31** | **2.42** | **10.5** | **5.31** | **5.6** | **393** | **312** | **175** |
| 5 | *0.9–1.8* | |  | *>50* |  |  |  |  |  | *1.38–3.08* | |  | *80–330* | |  |
|  | **0.76** | **1.3** | NR | **52** | NR | NR | **2.27** | **2.3** | NR | **4.24** | **4.56** | NR | **473** | **385** | NR |
| 6 | *1.0–1.8* | | |  | | | *2.2–2.6* | | | *1.3–9.3* | | | *75–400* | | |
|  | **0.56** | **0.9** | **0.6** | **35** | **55.7** | **37** | **2.37** | **2.46** | **2.31** | **9.7** | **5.9** | **8.3** | **610** | **251** | **191** |
| 7 | *0.74–1.55* | | *0.8–1.5* | *>50* | |  | *2.25–2.74* | *2.25–2.74* | *2.2–2.6* | *1.1–6.9* | | *1.1–6.9* | *63–316* | | *30–390* |
|  | **0.59** | **0.76** | **0.61** | **40** | **53** | NR | **2.22** | **2.26** | **2.3** | **18.7** | **21.3** | **10.3** | **604** | **179** | **176** |
| 8 | *0.97–1.94* | | *0.74–1.55* | *>50* | | | *2.25–2.74* | | | *1.1–6.9* | | | *38–123* | | |
|  | **0.54** | **1.22** | **0.51** | **17** | **51** | **38** | **2.30** | **2.24** | **2.17** | **13** | **11.3** | **9.2** | **542** | **113** | **104** |
| Individual values at each stage of treatment are available; where several values were available, “before” values are those closest to starting burosumab, “during” are values closest to the end of burosumab treatment, and “after” values are the furthest after stopping treatment.  Normal ranges are shown above each case study in *italics* (where reported).  ALP, alkaline phosphatase; NR, not reported; PTH, parathyroid hormone. | | | | | | | | | | | | | | | |
